# Identification of Potential Core Genes Associated With the Progression of Stomach Adenocarcinoma Using Bioinformatic Analysis

**DOI:** 10.3389/fgene.2020.517362

**Published:** 2020-10-22

**Authors:** Biao Yang, Meijing Zhang, Tianhang Luo

**Affiliations:** ^1^Department of General Surgery, Changhai Hospital, Second Military Medical University, Shanghai, China; ^2^Department of Oncology, Changhai Hospital, Second Military Medical University, Shanghai, China

**Keywords:** stomach adenocarcinoma, gene profiling, biomarker, differentially expressed genes, bioinformatical analysis

## Abstract

**Purpose:**

Stomach adenocarcinoma (STAD) is one of the most frequently diagnosed cancer in the world with both high mortality and high metastatic capacity. Therefore, the present study aimed to investigate novel therapeutic targets and prognostic biomarkers that can be used for STAD treatment.

**Materials and Methods:**

We acquired four original gene chip profiles, namely GSE13911, GSE19826, GSE54129, and GSE65801 from the Gene Expression Omnibus (GEO). The datasets included a total of 114 STAD tissues and 110 adjacent normal tissues. The GEO2R online tool and Venn diagram software were used to discriminate differentially expressed genes (DEGs). Gene ontology (GO) and Kyoto Encyclopedia of Gene and Genome (KEGG) enriched pathways were also performed for annotation and visualization with DEGs. The STRING online database was used to identify the functional interactions of DEGs. Subsequently, we selected the most significant DEGs to construct the protein-protein interaction (PPI) network and to reveal the core genes involved. Finally, the Kaplan-Meier Plotter online database and Gene Expression Profiling Interactive Analysis (GEPIA) were used to analyze the prognostic information of the core DEGs.

**Results:**

A total of 114 DEGs (35 upregulated and 79 downregulated) were identified, which were abnormally expressed in the GEO datasets. GO analysis demonstrated that the majority of the upregulated DEGs were significantly enriched in collagen trimer, cell adhesion, and identical protein binding. The downregulated DEGs were involved in extracellular space, digestion, and inward rectifier potassium channel activity. Signaling pathway analysis indicated that upregulated DEGs were mainly enriched in receptor interaction, whereas downregulated DEGs were involved in gastric acid secretion. A total of 80 DEGs were screened into the PPI network complex, and one of the most important modules with a high degree was detected. Furthermore, 10 core genes were identified, namely COL1A1, COL1A2, FN1, COL5A2, BGN, COL6A3, COL12A1, THBS2, CDH11, and SERPINH1. Finally, the results of the prognostic information further demonstrated that all 10 core genes exhibited significantly higher expression in STAD tissues compared with that noted in normal tissues.

**Conclusion:**

The multiple molecular mechanisms of these novel core genes in STAD are worthy of further investigation and may reveal novel therapeutic targets and biomarkers for STAD treatment.

## Introduction

Stomach adenocarcinoma (STAD) is one of the common malignant tumors, which accounts for high mortality and high incidence worldwide, notably in East Asia. In China alone, approximately 3,804,000 new cancer cases were diagnosed and 2,296,000 cancer deaths were reported in 2015 (Chen et al., 2018). Among them, the incidence and mortality of STAD ranked third (Bray et al., 2018). Although the gastroscopy and diagnostic techniques have made significant improvements in the treatment options of STAD, the overall survival rate for STAD patients remains unfavorable. According to the latest report, the 5-year survival rate for STAD is estimated to be approximately 10% (Chen et al., 2016). STAD is a complicated and gradual process and several genetic and environmental factors play important roles in its pathogenesis. Some of these risk factors, such as *H*. *pylori* infection, diet, smoking, chemical exposure, alcohol consumption, and exercise can also influence the development of STAD (Karimi et al., 2014). Cumulative evidence has shown that genetic factors, such as Glutathione S-transferase M1 (GSTM1)-null phenotypes and variants in the E-cadherin (CDH1), interleukin-17 (IL-17) and interleukin-10 (IL-10) contribute to the development of STAD (Meng et al., 2014; Long et al., 2015; Alvarez-Escola et al., 2019; Gao et al., 2019). Currently, numerous studies have focused on studying the mechanisms of STAD and several considerable improvements have been made in the efficacy of the clinical therapeutic methods. However, the lack of tumor-sensitive biomarkers that can be used early is considered to lead to poor prognosis. Therefore, it is essential to understand the pathogenesis and identification of novel promising prognostic biomarkers for individualized therapies, which can be beneficial in the improvement of life and survival of STAD.

In recent years, gene expression microarray and gene chip detection techniques have increased dramatically and biomedical research is commonly used to screen differentially expressed genes (DEGs) in a given organism and to identify prospective biomarkers for early diagnosis and advanced treatment of tumors (Vogelstein et al., 2013). The Gene Expression Omnibus (GEO) profiles and the Cancer Genome Atlas (TCGA) are public databases that have accumulated a large amount of core chipdata on the association between genes and diseases at the gene level (Petryszak et al., 2014). Therefore, large amounts of gene expression profiles and prognostic biomarkers can in theory be identified for STAD. Significant improvement has been made in the field of bioinformatic research on STAD in recent years (Liu et al., 2018; Pectasides et al., 2018; Chu et al., 2019). Nevertheless, the results are distinct or limited due to independent sample heterogeneity. To overcome these disadvantages, we adopted the methods of integrating bioinformatics with gene chip techniques.

In the present study, we obtained four original gene chip profiles, namely GSE13911, GSE19826, GSE54129, and GSE65801 from GEO. The datasets included a total of 114 STAD tissues and 110 adjacent normal tissues. The GEO2R online tool and Venn diagram software were used to discriminate DEGs. Gene ontology (GO) and Kyoto Encyclopedia of Gene and Genome (KEGG) enriched pathways were also performed for annotation and visualization with DEGs. The STRING online database was used to identify the functional interactions of DEGs. Subsequently, the most significant DEGs were selected to construct the protein-protein interaction (PPI) network and to reveal the core genes. Finally, the prognostic information was assessed for the core DEGs using the Kaplan-Meier Plotter online database and the Gene Expression Profiling Interactive Analysis (GEPIA). Due to its comprehensive analysis, the present study is one of the few to gather multiple databases regarding STAD. In conclusion, it can be deduced that the core DEGs and the enriched pathways in STAD may aid the screening and the identification of novel biomarkers and treatment targets of STAD in the future.

## Materials and Methods

### Microarray Data Information

The four gene chip profiles GSE13911, GSE19826, GSE54129, and GSE65801 containing information on STAD and adjacent normal tissues (ANT) were obtained from NCBI-GEO. The GSE13911, GSE19826, and GSE54129 were based on the GPL570 platforms, whereas GSE65801 was based on GPL14550. The GSE13911, GSE19826, GSE54129, and GSE65801 contained 38STAD and 31ANT, 12STAD and 15ANT, 111STAD and 21ANT, and 32STAD and 32ANT, respectively.

### Data Preprocessing of DEGs

The GEO2R online tools (Davis and Meltzer, 2007) were used to distinguish DEGs between stomach tumors and adjacent normal tissues by the cut-off criteria of adjusted *P* < 0.05 and |log2FC| > 1.5. Subsequently, the Venn software was used online to identify the original data among the four datasets and to reveal the commonly encountered DEGs.

### GO and Pathway Enrichment Analysis

Gene ontology (Ashburner et al., 2000) is a tool used to identify genes and proteins and to reveal the biological property of the chip database. KEGG (Kanehisa and Goto, 2000) is a collection of databases dealing with genomes and biological pathways. GO and KEGG analyses were used by the DAVID (Huang da et al., 2009), an online bioinformatic resource that can afford tools for several gene functions, such as DEG enrichment. The cut-off criterion was *P* < 0.05.

### PPI Network and Module Analysis

Initially, the search Tool for the Retrieval of Interacting Genes (STRING^1^) (Szklarczyk et al., 2015) was used to evaluate the PPI information. Secondly, Cytoscape (Shannon et al., 2003) was used to construct the potential association between these candidate DEGs. Finally, the Molecular Complex Detection (MCODE) software was used to screen the modules of the PPI network according to degree cutoff = 2, Depth = 100, k-core = 2, and node score cutoff = 0.2.

### Core Gene Analysis

The Kaplan-Meier Plotter online database was used to assess the overall survival of the core genes. GEPIA (Tang et al., 2017) was used to determine the expression levels related to the core genes. GEPIA is an online tool that can achieve characteristic functionalities based on TCGA and GTEx data. The hazard ratio (HR) with 95% confidence intervals and log-rank P value were computed and plotted.

## Results

### Identification of DEGs in STAD

**FIGURE 1:**
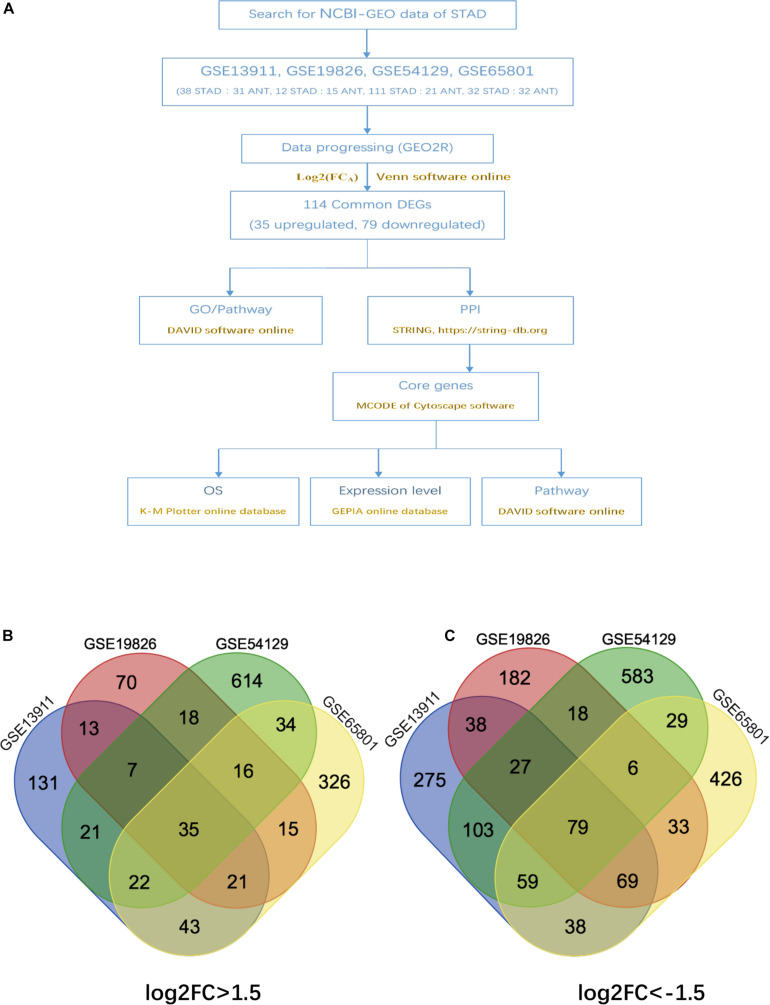
Authentication of 114 common DEGs in the four datasets (GSE13911, GSE19826, GSE54129, and GSE65801) through venn diagrams software (available online at: http://bioinformatics.psb.ugent.be/webtools/venn/). Different color meant different datasets. **(A)** Overall diagram of the study. **(B)** 35 DEGs were up-regulated in the four datasets (log2FC > 1.5). **(C)** 79 DEGs were down-regulated in the four datasets (log2FC > –1.5).

The overall design of this study is illustrated in Figure 1A. 4 gene expression array datasets were obtained from the GEO database as follows: GSE13911, GSE19826, GSE54129, and GSE65801, respectively (Table 1). Following screening of the data with GEO2R online tools with the cut-off criterion of adjusted *P* < 0.05 and |log2FC| > 1.5, 1,294, 899, 2,419, and 1,734 DEGs were screened from the four expression profile data, respectively. The volcano plot of the DEGs depending on FCs was displayed in Supplementary Figure 1. Finally, the commonly expressed 114 DEGs, including 35 upregulated and 79 downregulated genes were identified in the STAD tissues compared with the non-tumor samples via the Venn diagram software in the four datasets (Table 2 and Figures 1B,C).

**TABLE 1:** The detailed information of the four GEO datasets.

ID	Tissue	Platform	Normal	Tumor
GSE13911	STAD	GPL570	31	38
GSE19826	STAD	GPL571	15	12
GSE54129	STAD	GPL572	21	111
GSE65801	STAD	GPL14550	32	32

**TABLE 2:** All 114 commonly differentially expressed genes (DEGs) were detected from four profile datasets, including 79 downregulated genes and 35 upregulated genes in the STAD tissues compared to normal STAD tissues.

DEGs	Gene names									
Uprcgulated	ADAM 12	IGF2BP3	COL1A1	FNDC1	CST1	FN1	PRRX1	COL5A2	HOXA10	SPP1
	SFRP4	CDH11	BGN	COL8A1	ASPN	SERPINH1	FAP	INHBA	FSCN1	BMP1
	THBS2	NID2	MFAP2	WISP1	Sum	RARRES1	COL6A3	CLDN1	COL10A1	PMEPA1
	CTHRC1	EPHB2	COL1A2	COL12A1	SPOCK1					
Downregulated	LDHD	MAL	ADH7	ZBTB7C	LIPF	B4GALNT3 FM05	roc	TMED6	SULT1B1	
	FBP2	CAPN9	VSIG1	CWH43	PDIA2	CYP2C18	CA2	B3GNT6	SCNN1G	CLDN18
	AKR1B10	PKIB	CA9	SCGB2A1	LOC400043 ALDH3A1	GATA5	KCNE2	PSAPL1	FBXL13	
	PTPRZ1	ESRRG	GCNT2	TMPRSS2	ARHGEF37 FUT9	ATP4B	SOSTDC1	KLKU	GKN2	
	ATP4A	AKR7A3	SSI	CXCL17	CAPN13	RDH12	SLC26A9	ENPP6	PSCA	BEX5
	UGT2B15	CPA2	TFF2	SPINK2	TCN1	C16orf89	VSTM2A	RORC	KCNJ16	HYAL1
	KIAA1324	RAB27B	SCNN1B	LYPD6B	HOMER2	GIF	SSTR1	MUC5AC	KCNJ15	TFF1
	GKN1	DPCR1	HPGD	CNTN3	MUC6	ALDH1A1	ACER2	VSIG2	ASCL1	

### DEGs, GO, and KEGG Pathway Analysis in STAD

**FIGURE 2:**
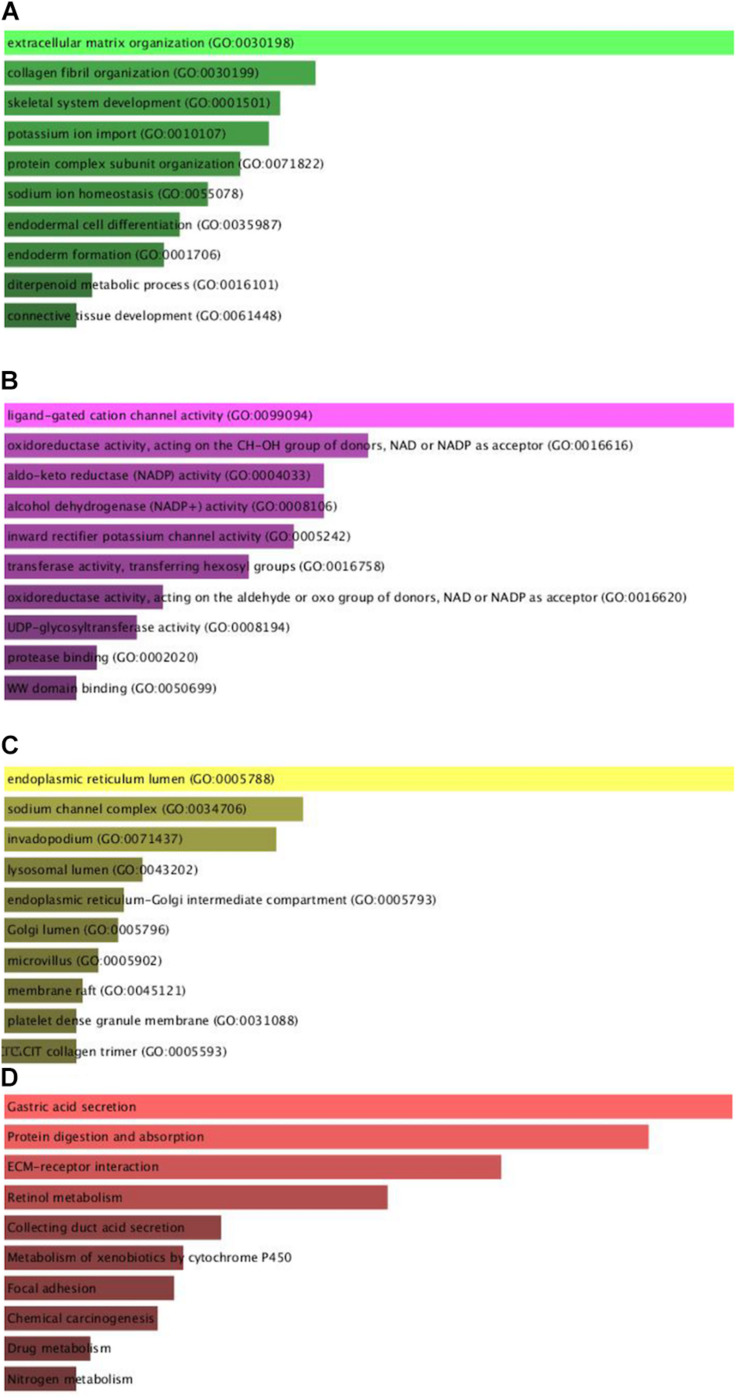
Gene Ontology enrichment and KEGG pathway analysis of the differentially expressed genes. **(A–D)** The numbers of enriched according to the **(A)** biological process, **(B)** molecular function, **(C)** cellular component categories, and **(D)** KEGG pathway analysis.

To comprehend the DEG functional levels, the online biological tool DAVID6.8 was performed using the GO analysis with a significance threshold of *P* < 0.05. The results of the 34 DEGs in the GO terms of the categories were divided into three groups as follows: biological process (BP), cellular component (CC), and molecular function (MF). As indicated in Table 3, the CC of overexpressing DEGs were mainly enriched in collagen trimer, proteinaceous extracellular matrix, extracellular space, extracellular exosome, and extracellular region; the downregulated DEGs were involved in the extracellular space, apical plasma membrane, extracellular exosome, and anchored component of membrane and lysosome. The BP of the overexpressing DEGs was mainly enriched in cell adhesion, endodermal cell differentiation, collagen fibril organization, cellular response to amino acid stimulus, and skeletal system development. The downregulated DEGs were involved in digestion, cellular aldehyde metabolic process, xenobiotic metabolic process, oxidation-reduction process, and potassium ion import. The MF of the overexpressing DEGs were mainly enriched in identical protein binding, extracellular matrix structural constituent, protein binding, calcium ion binding, and platelet-derived growth factor binding; the down-regulated DEGs were involved in inward rectifier potassium channel activity, benzaldehyde dehydrogenase (NAD+) activity, hydrogen:potassium-exchanging ATPase activity, retinal dehydrogenase activity, and ligand-gated sodium channel activity. In general, the GO terms of the top 10 were displayed in Figures 2A–C according to the *P*-value (Supplementary Table 2).

**TABLE 3:** Gene ontology analysis of differentially expressed genes in STAD.

Expression	Category	Term	Count	*P*-value	FDR
Upregulated	GOTERM_BP_DIRECT	GO:0007155∼cell adhesion	7	8.38E-07	0.001059856
	GOTERM_BP_DIRECT	GO:0035987∼endodermal cell differentiation	4	1.68E-05	0.021196552
	GOTERM_BP_DIRECT	GO:0030199∼collagen fibril organization	4	2.35E-05	0.029671995
	GOTERM_BP_DIRECT	GO:0071230∼cellular response to amino acid stimulus	3	0.003303913	4.09798155
	GOTERM_BP_DIRECT	GO:()001501-skeletal system development	3	0.004586779	5.647054851
	GOTERM_CC_DIRECT	GO:0005581-collagen trimer	7	1.31E-09	135E-06
	GOTERM_CC_DIRECT	GO:0005578∼proteinaceous extracellular matrix	8	5.16E-08	5.29E-05
	GOTERM_CC_DIRECT	GO:0005615∼extracellular space	12	1.21E-06	0.001236783
	GOTERM_CC_DIRECT	GO:0070062∼extrdcellular exosome	12	0.002224851	2.258522618
	GOTERM_CC_DIRECT	GO:0005576∼extracellular region	6	0.00306613	3.100466564
	GOTERM_MF_DIRECT	GO:0042802∼identical protein binding	4	0.002087486	1.983628784
	GOTERM_MF_DIRECT	GO:0005201-extracellular matrix structural constituent	3	0.002236986	2.124328943
	GOTERM_MF_DIRECT	GO:0005515∼protein binding	4	0.003812268	3.59592875
	GOTERM_MF_DIRECT	GO:0005509∼calcium ion binding	6	0.003982864	3.75410086
	GOTERM_MF_DIRECT	GO:0048407∼platelet-derived growth factor binding	2	0.005716885	5.348701785
Downregulated	GOTERM_BP_DIRECT	GO:0007586∼digestion	8	6.67E-09	9.09E-06
	GOTERM_BP_DIRECT	GO:0006081∼cellular aldehyde metabolic process	4	1.07E-05	0.014571778
	GOTERM_BP_DIRECT	GO:0006805∼xenobiotic metabolic process	6	1.69E-05	0.023039718
	GOTERM_BP_DIRECT	GO:(X)55114∼oxidation-reduction process	12	2.71 E-05	0.036936867
	GOTERM_BP_DIRECT	GO:0010107∼potassium ion import	4	2.02E-04	0.27485333
	GOTERM_CC_DIRECT	GO:0005615∼extracellular space	20	1.49E-06	0.001568694
	GOTERM_CC_DIRECT	GO:0016324∼apical plasma membrane	6	0.006968923	7.094816498
	GOTERM_CC_DIRECT	GO:0070062∼extracellular exosome	21	0.008418185	8.511744286
	GOTERM_CC_DIRECT	GO:0031225∼anchored component of membrane	4	0.011341676	11.31066102
	GOTERM_CC_DIRECT	GO:0005764∼lysosome	5	0.014100077	13.88022987
	GOTERM_MF_DIRECT	GO:0005242∼inward rectifier potassium channel activity	3	0.002416557	2.873134203
	GOTERM_MF_DIRECT	GO:0018479∼benzaldehyde dehydrogenase (NAD +) activity	2	0.007332264	8.48537793
	GOTERM_MF_DIRECT	GO:0008900∼hydrogen:potassium-exchanging ATPase activity	2	0.010978534	12.45446088
	GOTERM_MF_DIRECT	GO:0001758∼retinal dehydrogenase activity	2	0.025432302	26.68434583
	GOTERM_MF_DIRECT	GO:0015280∼ligand-gated sodium channel activity	2	0.029013148	29.86504406

Furthermore, to distinguish the potential pathway of DEGs, we used KEGG pathway enrichment analyses. As indicated in Figure 2D and Table 4, the results demonstrated that upregulation of DEGs was mainly enriched in receptor interaction, protein digestion, and absorption and focal adhesion. The downregulated DEGs were involved in gastric acid secretion, retinol metabolism, and drug metabolism-cytochrome P450.

**TABLE 4:** KEGG pathway analysis of differentially expressed genes in STAD.

Pathwav ID	Description	Count	*P*-value	Genes
hsa04971	Gastric acid secretion	7	2.00E-05	KCNJ16, KCNJ15, ATP4A, ATP4B, KCNE2, CA2, and SST
hsa04512	ECM-receptor interaction	7	5.46E-05	COL6A3, COL1A2, COL1 Al, THBS2, COL5A2, SPP1, and FN1
hsa04974	Protein digestion and absorption	7	5.83E-05	COL6A3, COL1A2, CPA2, COL12A1, COL1A1, COL5A2, and COL10A1
hsa00830	Retinol metabolism	5	0.001520866	ALDH1A1, RDH12, CYP2C18, ADH7, and UGT2B15
hsa04510	Focal adhesion	7	0.005227439	COL6A3, COL1A2, COL1 Al, THBS2, COL5A2, SPP1, and FN1
hsa00982	Drug metabolism – cytochrome P450	4	0.016001409	FM05, ADH7, UGT2B15, and ALDH3A1
hsa04966	Collecting duct acid secretion	3	0.018727092	ATP4A, ATP4B, and CA2
hsa00980	Metabolism of xenobiotics by cytochrome P450	4	0.020028855	AKR7A3, ADH7, UGT2B15, and ALDH3A1
hsa05204	Chemical carcinogenesis	4	0.024566639	CYP2C18, ADH7.UGT2B15, and ALDH3A1

### DEG PPI and Modular Analysis

**FIGURE 3:**
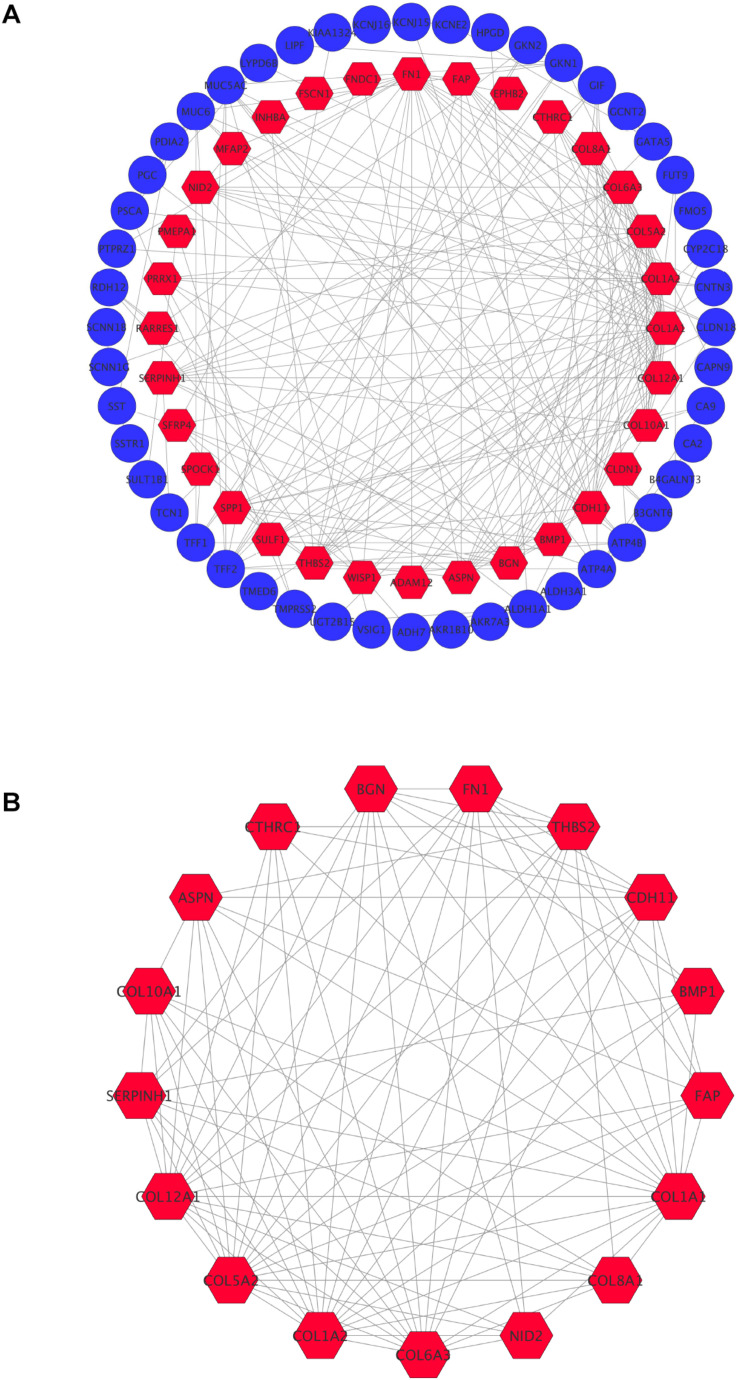
Common DEGs PPI network constructed by STRING online database and Module analysis. **(A)** There were a total of 80 DEGs in the DEGs PPI network complex. The nodes meant proteins, the edges meant the interaction the proteins, blue circule meant down-regulated DEGs, and red hexagons meant up-regulated DEGs. **(B)** Module analysis via Cytoscope software (degree cutoff = 2, node score cutoff = 0.2, k-core = 2, and max Depth = 100).

In order to achieve core candidate gene and vital gene modules in STAD, PPI network analysis was performed. A total of 80 DEGs were screened into the PPI network complex, involving 80 nodes and 215 edges, and the remaining 34 DEGs were not screened (Figure 3A). According to Cytoscape, 14 central node genes were identified depending on the criteria of the edge degree ≥ 10 (Table 5 and Supplementary Table 1). According to the edge degree rank, the 10 core genes were COL1A1, COL1A2, FN1, COL5A2, BGN, COL6A3, COL12A1, THBS2, CDH11, and SERPINH1. Furthermore, we used the MCODE plug-in to screen the highest degree module in the PPI network. The results of the analysis revealed that the highest degree module contained 17 nodes and 92 edges (Figure 3B).

**TABLE 5:** The central node genes in the PPI network were identified based on the filtering degree ≥10.

Node gene	Degree
COL1A1	24
COL1A2	21
FN1	20
COL5A2	18
BGN	16
COL6A3	16
COL12A1	15
THBS2	15
CDH11	12
SERPINH1	11
COL10A1	11
TFF2	11
ASPN	11
MUC5AC	10

### Core Gene Analysis

**FIGURE 4:**
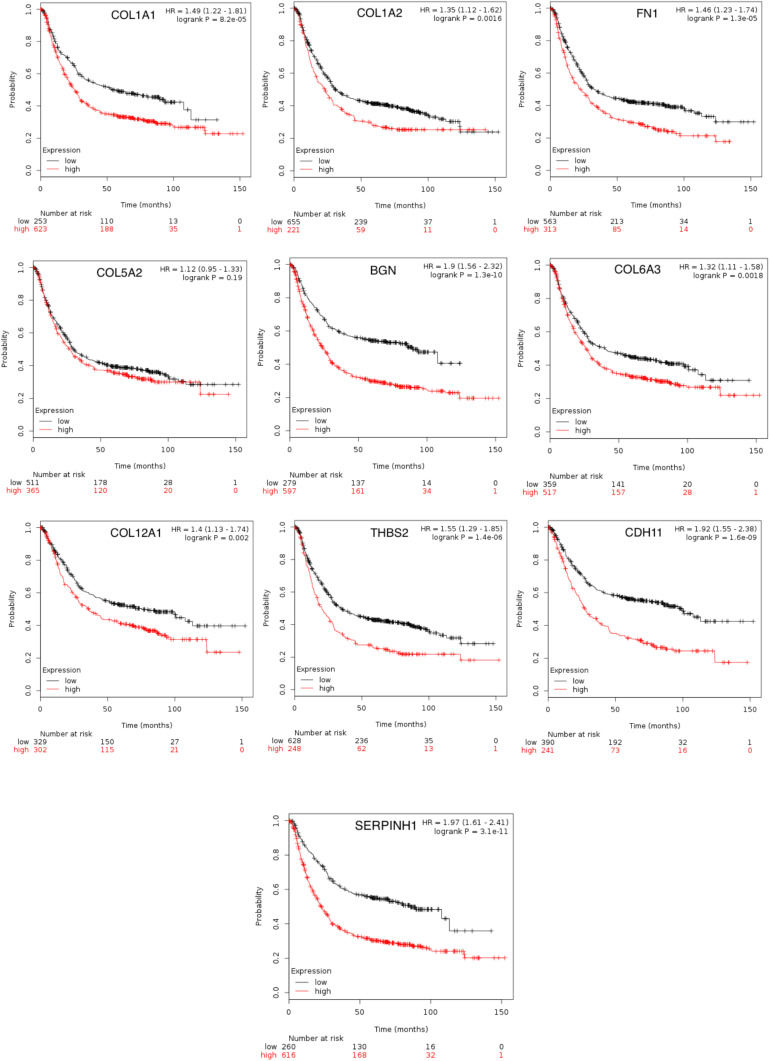
The prognostic information of the 10 core genes. Kaplan-Meier plotter online tools were used to identify the prognostic information of the 10 core genes and genes had a significantly worse survival rate (*P* < 0.05).

**FIGURE 5:**
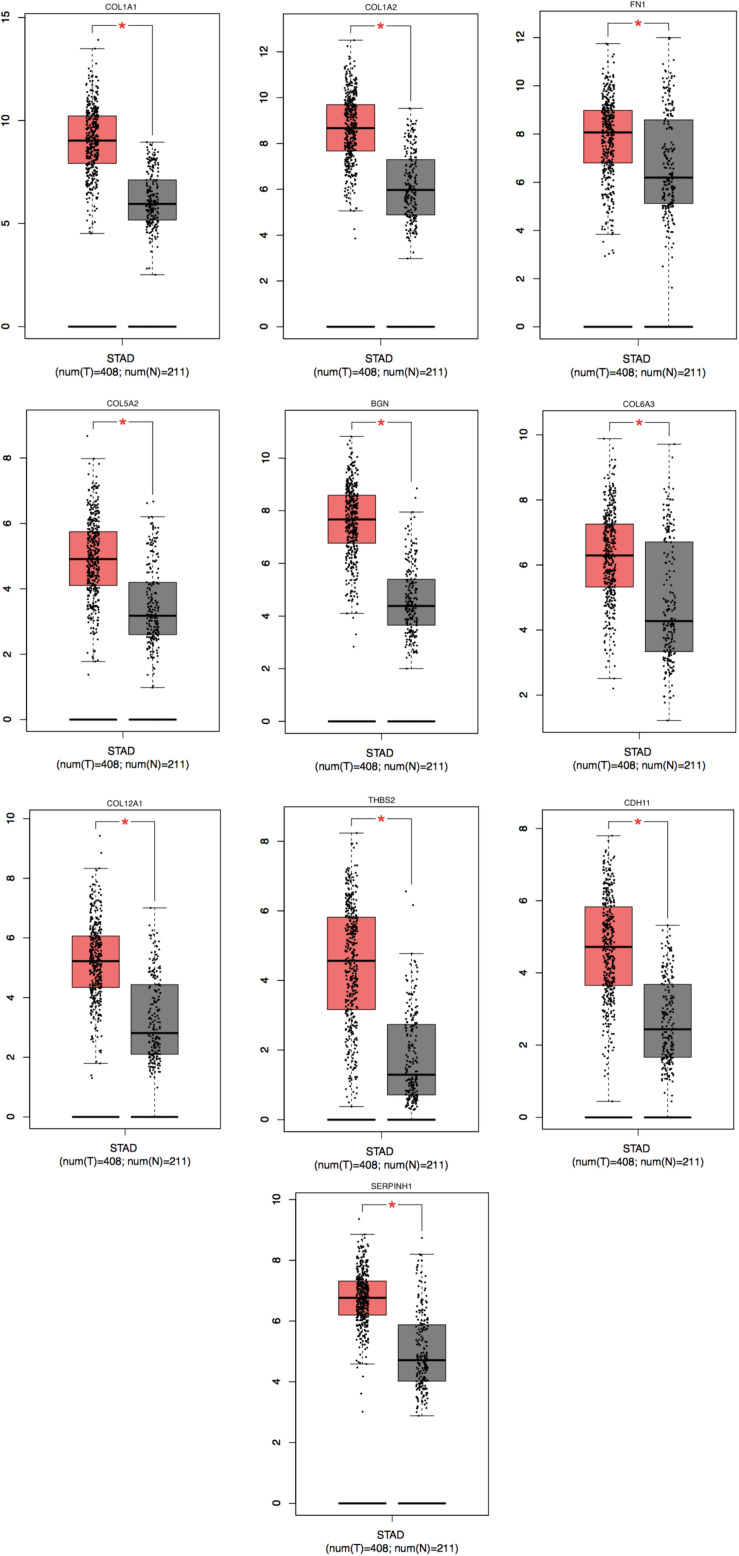
Significantly expressed 10 genes in STAD cancer patients compared to healthy people. To future identify the genes’ expression level between STAD cancer and normal people, 10 genes which were related with poor prognosis were analyzed by GEPIA website. A total of 10 genes significant expression level in STAD specimen compared to normal specimen (*P < 0.05). Red color means tumor tissues and gray color means normal tissues.

**FIGURE 6:**
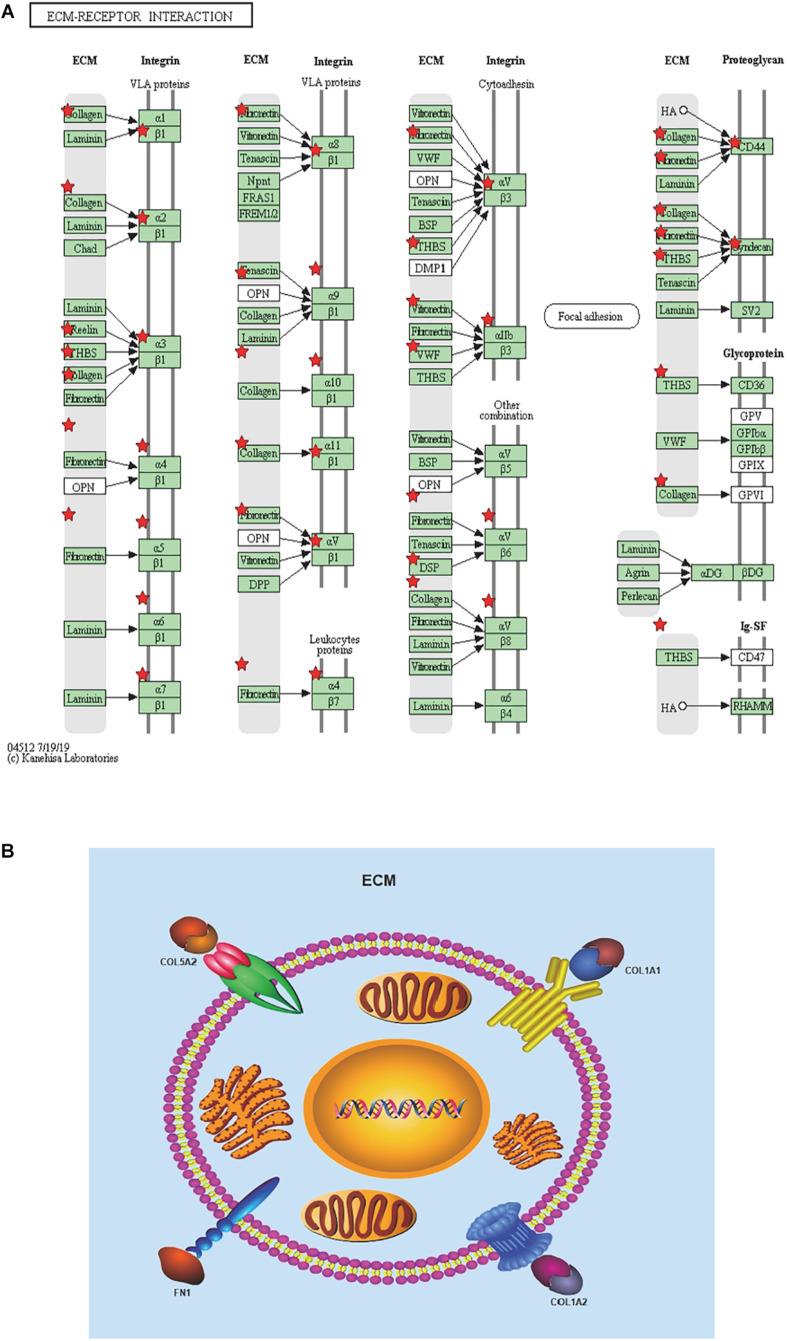
Re-analysis of 10 selected genes by KEGG pathway enrichment. **(A)** 10 high expressed genes in STAD tissues with poor prognosis were re-analyzed by KEGG pathway enrichment and they were significantly enriched in ECM-receptor interaction. List 10 genes are shown in red^``*”^. **(B)** Presumed patterns of changes in the ECM-receptor interaction pathway of the four most expressed genes.

To achieve the 10 core-gene survival data, we performed Kaplan-Meier curves to analyze the overall survival. The results indicated that all 10 core genes exhibited a prominent prognosis for STAD patients (*P* < 0.05, Figure 4). Subsequently, we analyzed the expression status of these genes using the GEPIA. The results indicated that all 10 core genes exhibited significantly higher expression in the STAD tissues compared with those of the normal tissues (*P* < 0.05, Figure 5). Subsequently, we re-analyzed all 10 core genes associated with poor survival in STAD by KEGG pathway enrichment. The results of the re-analysis indicated that six genes (COL6A3, COL1A2, COL1A1, THBS2, COL5A2, and FN1) were significantly enriched in the extracellular matrix-receptor (ECM-receptor) interaction (*P* < 0.05, Table 6 and Figure 6).

**TABLE 6:** Re-analysis of 10 selected genes via the KEGG pathway enrichment.

Pathway ID	Term	Count	*P*-value	Genes	FDR
XU04512	ECM-receptor interaction	6	1.12E-09	COL6A3, COL1A2, COL1 Al, THBS2, COL5A2, FN1	4.28E-07
xtrO4510	Focal adhesion	6	1.19E-07	COL6A3, COL1A2, COL1 Al, THBS2, COL5A2, FN1	4.53E-05

## Discussion

Stomach adenocarcinoma is one of the most frequently diagnosed cancers in the world with both high mortality and high metastatic capacity (Siegel et al., 2017). Certain genes have been shown to play an important role in STAD. It has been reported that CDH1 may be used in identifying families with high risk of cancer as well as aiding the design of chemopreventive programs that are focused at high-risk subgroups (van der Post et al., 2015). It is well known that the GSTM1-null phenotype can increase significantly the risk of STAD (Darazy et al., 2011; Qiu et al., 2011; García-González et al., 2012; Jing et al., 2012). In spite of a large number of studies examining STAD, its molecular mechanism has not been satisfactory explained due to the limited number of stable and effective markers. The main reason is that previous studies were too narrow. Therefore, multiple cohort types of research regarding effective molecular biomarkers are required for STAD prevention, diagnosis and treatment.

In the present study, the identification of more effective molecular biomarkers for STAD was performed by merging four profile datasets (GSE13911, GSE19826, GSE54129, and GSE65801). Bioinformatic analysis was performed and resulted in the identification of 193 STAD and 99ANT genes. Subsequently, the commonly identified 114 DEGs included 35 upregulated and 79 downregulated genes in STAD tissues compared to those noted in the non-tumor samples, which were identified by the Venn diagram software in the four datasets. For the purpose of an in-depth understanding of the DEG functional levels, we used the GO function and KEGG pathway to analyze these DEGs. Subsequently, PPI network analysis was used to identify these DEGs based on Cytoscape software and the online database STRING. A total of 80 DEGs were screened by the PPI network complex, involving 215 edges. The highest degree module was screened from the PPI by the MCODE plug-in. Eventually, 10 core DEGs were identified according to the edge degree rank in the PPI network complex and the results of the survival analysis demonstrated that the patients with aberrant expression of DEGs exhibited a significantly lower survival for STAD patients. In addition, we re-analyzed all 10 core genes with poor survival in STAD by KEGG pathway enrichment. The results of the re-analysis indicated that the six genes (COL6A3, COL1A2, COL1A1, THBS2, COL5A2, and FN1) were significantly enriched in the ECM-receptor interaction. Among these genes, COL1A1, COL1A2, FN1 and COL5A2 were considered as perspective effective targets that play prominent roles in the development and recurrence of the tumor, including STAD.

COL1A1 and COL1A2 are the genes, which encode the pro-alpha chains of type I collagen whose triple helix comprises two alpha 1 chains and one alpha 2 chain. It has been reported that the potential of the COL1A1 gene structure and intron variation for common bone-related diseases can be determined by comparative vertebrate evolutionary analyses of type I collagen (Stover and Verrelli, 2011). COL1A1 can be used as a new therapeutic marker and a target for hepatocellular carcinogenesis (Ma et al., 2019). Another study demonstrated that COL1A2 may affect proliferation, migration, and invasion of colorectal cancer cells (Yu et al., 2018). Omar Ret al., reported that COL1A2 affects cell migration of fibrosarcoma and chondrosarcoma by acting on TBX3 (Omar et al., 2019). Several studies have shown that COL1A1/2 plays a huge role in osteogenesis (Pollitt et al., 2006; Sato et al., 2016; Wang et al., 2019; Zhytnik et al., 2019). COL1A1 and COL1A2 have been shown to play an important prognostic role in STAD (Tamilzhalagan et al., 2017; Shi et al., 2019; Li J. et al., 2020). Recently Wang et al., reported that COL1A1 suppressed the invasion and migration of STAD cells by combining with miR-129-5p (Wang and Yu, 2018). Furthermore, COL1A2 was reported to suppress STAD cell invasion, and migration via regulation of the PI3k-Akt signaling pathway (Ao et al., 2018).

FN1, encodes fibronectin, a glycoprotein present in a soluble dimeric form in plasma and in a dimeric or multimeric form at the cell surface and in the extracellular matrix. Cai et al. demonstrated that the low expression of FN1 in colorectal cancer could significantly inhibit the growth and metastasis of tumor cells (Cai et al., 2018). Cadoff et al., demonstrated specific mechanistic insights into the cellular effects of a novel FN1 variant associated with a spondylometaphyseal dysplasia (Cadoff et al., 2018). Liu et al., indicated that the low expression of NEAT1 could affect the radioactive iodine resistance by the miR-101-3p/FN1/PI3K-AKT signaling pathway in papillary thyroid carcinoma cells (Liu et al., 2019). Gene expression database research demonstrated that FN1 could be used as a new marker of radiation resistance for head and neck cancer (Amundson and Smilenov, 2010; Zhan et al., 2018). In addition, FN1 is often detected in STAD tissues and cell lines and its abnormal expression is closely associated with the invasion and metastasis of STAD (Xu et al., 2014; Arita et al., 2016; Sun et al., 2020). Moreover, it has been reported that FN1 combined with microRNA-200c can inhibit the migration and invasion of STAD cells (Zhang et al., 2017).

COL5A2 encodes an alpha chain for one of the low abundance fibrillar collagens. Fibrillar collagen molecules are trimers that can be composed of one or more types of alpha chains. Yang et al., indicated that the decrease of COL5A2 expression could induce femoral head necrosis (Yang et al., 2018). Park et al., demonstrated that abnormal expression of COL5A2 may lead to new abnormalities in skin and adipose tissue, which can further lead to the occurrence of aortic aneurysms and dissections (Park et al., 2017). Park et al., demonstrated that homozygosity and heterozygosity for null COL5A2 alleles produced embryonic lethality and a novel classic Ehlers-Danlos syndrome-related phenotype (Park et al., 2015). A retrospective analysis of bladder cancer gene expression data presented that COL5A2 in patients with bladder cancer and ischemic heart disease may possess important clinical significance (Azuaje et al., 2013; Meng et al., 2018; Zeng et al., 2018). Moreover, COL5A2 was considered a potential molecular marker in STAD using bioinformatic analysis (Li J. et al., 2020; Li Z. et al., 2020). However, a limited number of reports have been conducted on the mechanism of COL5A2 in STAD.

In the present study, we identified candidate biomarkers that may play a distinct clinical significance of STAD. These newly discovered core genes could be regarded as potential biomarkers to further explore the molecular mechanism and the prognostic effects of STAD. However, the present study contains certain limitations, which can be listed as follows: (1) the present study requires additional experiments to complement the bioinformatic analysis; (2) the basic characteristics of the tumor, such as gender, age, sample size, tumor grade and stage and main misleading outcomes were not considered in the present study; (3) although 4 datasets were included, no definitive results could be obtained. Therefore, subsequent studies should be employed to confirm the association between these core genes and STAD.

## Conclusion

In summary, the present study integrated four different microarray GEO datasets, and identified 114 DEGs, including 35 upregulated and 79 downregulated genes. Subsequently, we observed that four core genes (COL1A1, COL1A2, FN1, and COL5A2) exhibited the highest interaction degrees. The results of the analysis demonstrate that these four genes play prominent roles in the complicated and gradual process of STAD. However, the primary conclusions of the analysis require further confirmation by a series of clinical experiments. The multiple molecular mechanisms of these novel core genes in STAD may reveal novel therapeutic targets and biomarkers for STAD treatment.

## Data Availability Statement

Publicly available datasets were analyzed in this study. This data can be found here: GEO database (https://www.ncbi.nlm.nih.gov/geo), accession numbers: GSE13911, GSE19826, GSE54129, and GSE65801.

## Author Contributions

BY and TL designed the work and prepared the figures and tables. BY wrote the main manuscript text. MZ prepared the acquisition, analysis, and interpretation of data. Both authors contributed to the article and approved the submitted version.

## Conflict of Interest

The authors declare that the research was conducted in the absence of any commercial or financial relationships that could be construed as a potential conflict of interest.

## Footnotes

1 https://string-db.org
