# Supplementary material for: Identification of Potential Core Genes Associated With the Progression of Stomach Adenocarcinoma Using Bioinformatic Analysis
Source: Front Genet. 2020 Oct 22;11:517362. doi: 10.3389/fgene.2020.517362 (PMC7642829; doi:10.3389/fgene.2020.517362)
Supplement: Supplementary file 1 [file Data_Sheet_1.PDF]

Supplementary Table 1. Information of 10 core DEGs

Gene.symbol	group	adj.P.Val	$log2FC_A$
COL1A1	up	0.00501	2.66870
FN1	up	0.01844	1.48275
COL5A2	up	0.00034	1.70204
CDH11	up	0.00204	2.00854
BGN	up	0.00073	2.17694
SERPINH1	up	0.00025	2.55090
THBS2	up	0.00106	3.22939
COL6A3	up	0.00020	2.21399
COL1A2	up	0.00015	2.11779
COL12A1	up	0.00080	2.10952

Supplementary Table 2 Gene Ontology enrichment and KEGG pathway analysis of the differentially expressed genes.

A

Term	Adjusted P-value	Combined Score	Genes
extracellular matrix organization (GO:0030198)	1.65E-08	306.7507065	COL12A1;FN1;BGN;NID2;COL1A1;BMP1;COL1A2;ADAM12;COL5A2;MFAP2;SERPINH1;SPP1;COL8A1;COL10A1;COL6A3
collagen fibril organization (GO:0030199)	0.001431976	439.2168738	COL1A1;COL1A2;COL12A1;COL5A2;SERPINH1
skeletal system development (GO:0001501)	0.003033852	128.3733184	COL1A1;BMP1;COL1A2;HYAL1;COL12A1;CDH11;COL10A1;SULF1
potassium ion import (GO:0010107)	0.002906202	302.5663219	ATP4B;ATP4A;KCNE2;KCNJ15;KCNJ16
protein complex subunit organization (GO:0071822)	0.005483799	238.6254309	COL1A1;COL1A2;COL12A1;COL5A2;SERPINH1
sodium ion homeostasis (GO:0055078)	0.01115878	306.095387	ATP4B;ATP4A;SCNN1G;SCNN1B
endodermal cell differentiation (GO:0035987)	0.022413204	229.8623615	COL12A1;FN1;COL8A1;INHBA
endoderm formation (GO:0001706)	0.03157087	194.9589529	COL12A1;FN1;COL8A1;INHBA
diterpenoid metabolic process (GO:0016101)	0.237396448	88.81967526	AKR1B10;RDH12;ALDH1A1;ADH7
connective tissue development (GO:0061448)	0.325542972	130.216984	HYAL1;RORC;SULF1

B

Term	Adjusted P-value	Combined Score	Genes
ligand-gated cation channel activity (GO:0099094)	0.025844673	157.8766546	SCNN1G;KCNE2;SCNN1B;KCNJ15;KCNJ16
oxidoreductase activity, acting on the CH-OH group of donors, NAD or NADP as acceptor (GO:0016616)	0.081759539	89.09103	AKR7A3;AKR1B10;HPGD;RDH12;ADH7
aldo-keto reductase (NADP) activity (GO:0004033)	0.071609969	227.9547374	ALDH3A1;AKR7A3;AKR1B10
alcohol dehydrogenase (NADP+) activity (GO:0008106)	0.053707476	227.9547374	ALDH3A1;AKR1B10;RDH12
inward rectifier potassium channel activity (GO:0005242)	0.049921072	213.3062095	KCNE2;KCNJ15;KCNJ16
transferase activity, transferring hexosyl groups (GO:0016758)	0.04963724	73.09416693	FUT9;HYAL1;B3GNT6;UGT2B15;B4GALNT3
oxidoreductase activity, acting on the aldehyde or oxo group of donors, NAD or NADP as acceptor (GO:0016620)	0.060657652	167.8935025	ALDH3A1;AKR1B10;ALDH1A1
UDP-glycosyltransferase activity (GO:0008194)	0.064044727	86.72137596	HYAL1;B3GNT6;UGT2B15;B4GALNT3
protease binding (GO:0002020)	0.070961134	56.22307481	COL1A1;CST1;COL1A2;FAP;FN1
WW domain binding (GO:0050699)	0.066356657	136.5611079	SCNN1G;SCNN1B;PMEPA1

C

Term	Adjusted P-value	Combined Score	Genes
endoplasmic reticulum lumen (GO:0005788)	3.12E-06	83.11304485	COL1A1;COL1A2;COL12A1;COL5A2;SERPINH1;FN1;SPP1;COL8A1;COL10A1;COL6A3
sodium channel complex (GO:0034706)	0.001382294	233.0623223	SCNN1G;SCNN1B
invadopodium (GO:0071437)	0.002012448	183.138748	FAP;FSCN1
lysosomal lumen (GO:0043202)	0.012797079	26.91009455	HYAL1;GIF;BGN
endoplasmic reticulum-Golgi intermediate compartment (GO:0005793)	0.016689432	22.87645773	SERPINH1;FN1;TMED6
Golgi lumen (GO:0005796)	0.018118227	21.73110062	BGN;MUC5AC;MUC6
microvillus (GO:0005902)	0.023517997	31.6054726	GIF;FSCN1
membrane raft (GO:0045121)	0.029974989	15.64984872	SERPINH1;MAL;SULF1
platelet dense granule membrane (GO:0031088)	0.033428678	100.2460508	RAB27B
FACIT collagen trimer (GO:0005593)	0.033428678	100.2460508	COL12A1

D

Term	Adjusted P-value	Combined Score	Genes
Gastric acid secretion	6.78E-05	253.2114663	ATP4B;ATP4A;KCNE2;CA2;SST;KCNJ15;KCNJ16
Protein digestion and absorption	1.19E-04	193.7197062	COL1A1;CPA2;COL1A2;COL12A1;COL5A2;COL10A1;COL6A3
ECM-receptor interaction	7.22E-04	153.6671385	COL1A1;COL1A2;SPP1;FN1;COL6A3;THBS2
Retinol metabolism	0.002962817	134.267869	RDH12;UGT2B15;ALDH1A1;ADH7;CYP2C18
Collecting duct acid secretion	0.028662647	150.8912952	ATP4B;ATP4A;CA2
Metabolism of xenobiotics by cytochrome P450	0.042087242	67.98709336	ALDH3A1;AKR7A3;UGT2B15;ADH7
Focal adhesion	0.041401124	37.18754952	COL1A1;COL1A2;FN1;SPP1;COL6A3;THBS2
Chemical carcinogenesis	0.046351637	58.03718468	ALDH3A1;UGT2B15;ADH7;CYP2C18
Drug metabolism	0.112856305	37.45994831	ALDH3A1;UGT2B15;ADH7;FMO5
Nitrogen metabolism	0.125390116	114.6036474	CA2;CA9

(A-D) The numbers of enriched genes according to the (A) biological process, (B) molecular function, (C) cellular component categories (D) KEGG pathway analysis.

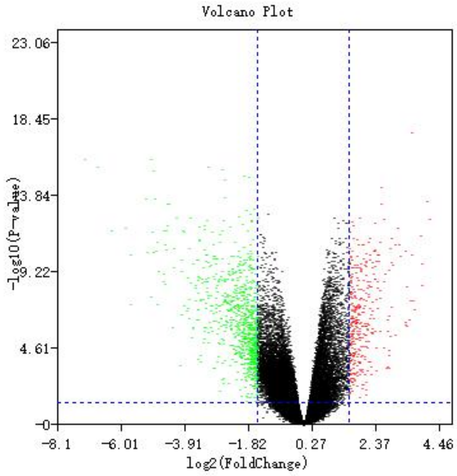

A GSE13911

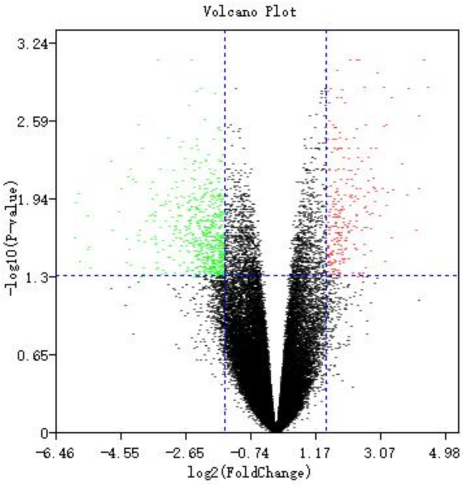

B GSE19826

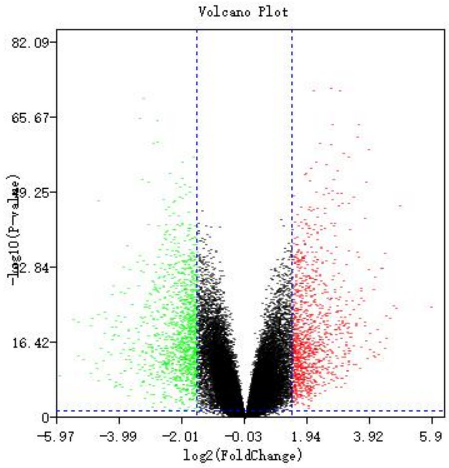

C GSE54129

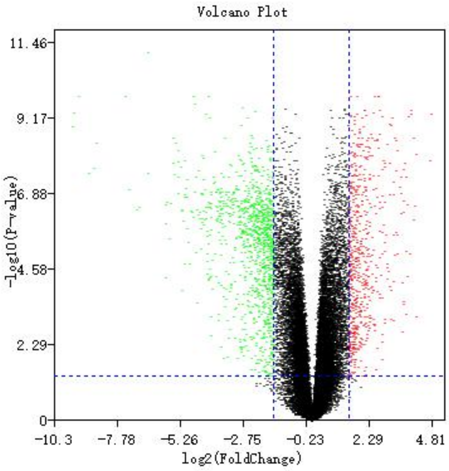

D GSE65801

Supplementary Fig. 1 Analysis of the differentially expression genes between the STAD and non-STAD using Volcano plots

Red and green points represent up-regulated and down-regulated genes. No differentially expressed genes in black points. (A) DEGs based on GSE13911. (B) DEGs based on GSE519826. (C) DEGs based on GSE54129. (D) DEGs based on GSE65801.
